# Geniposide Regulates Glucose-Stimulated Insulin Secretion Possibly through Controlling Glucose Metabolism in INS-1 Cells 

**DOI:** 10.1371/journal.pone.0078315

**Published:** 2013-10-22

**Authors:** Jianhui Liu, Lixia Guo, Fei Yin, Yonglan Zhang, Zixuan Liu, Yanwen Wang

**Affiliations:** 1 Chongqing Key Laboratory of Catalysis & Functional Organic Molecules, Chongqing Technology and Business University, Chongqing, China; 2 Chongqing Key Laboratory of Natural Medicine Research, Chongqing Technology and Business University, Chongqing, China; 3 Aquatic and Crop Resource Development, National Research Council of Canada, Charlottetown, Prince Edward Island, Canada; 4 College of Chemistry and Chemical Engineering, Chongqing University, Chongqing, China; Consiglio Nazionale delle Ricerche, Italy

## Abstract

Glucose-stimulated insulin secretion (GSIS) is essential to the control of metabolic fuel homeostasis. The impairment of GSIS is a key element of β-cell failure and one of causes of type 2 diabetes mellitus (T2DM). Although the K_ATP_ channel-dependent mechanism of GSIS has been broadly accepted for several decades, it does not fully describe the effects of glucose on insulin secretion. Emerging evidence has suggested that other mechanisms are involved. The present study demonstrated that geniposide enhanced GSIS in response to the stimulation of low or moderately high concentrations of glucose, and promoted glucose uptake and intracellular ATP levels in INS-1 cells. However, in the presence of a high concentration of glucose, geniposide exerted a contrary role on both GSIS and glucose uptake and metabolism. Furthermore, geniposide improved the impairment of GSIS in INS-1 cells challenged with a high concentration of glucose. Further experiments showed that geniposide modulated pyruvate carboxylase expression and the production of intermediates of glucose metabolism. The data collectively suggest that geniposide has potential to prevent or improve the impairment of insulin secretion in β-cells challenged with high concentrations of glucose, likely through pyruvate carboxylase mediated glucose metabolism in β-cells.

## Introduction

Type 2 diabetes mellitus (T2DM) is a heterogeneous, multifactorial, polygenic disease characterized by a defect in either insulin secretion and/or action that results in elevated circulating glucose [1]. Accumulating evidence has shown that in the early stage of T2DM, insulin secretion is increased to compensate for insulin resistance. However, increased insulin production and release, if continues for an extended time, gradually exhausts the pancreatic β cells and result in insulin deficiency eventually. Therefore, impairment of β cell function or cell death due to prolonged exposure to high glucose stimulations in insulin resistance is an important causative factor in the progression of insulin resistance towards T2DM [2,3]. It is indeed demonstrated that rodent β-cell function and survival are maintained in the presence of 10 mM of glucose but substantially impaired when exposed to 30 mM of glucose [4,5]. Similar phenomena have been seen in human diabetics as evidenced by findings that supraphysiological glucose concentrations are deleterious to β-cell function and survival, resulting in the alteration of the functional β-cell mass and contributing to the progressive worsening of glucose intolerance in T2DM patients [6–8]. Accordingly, it is hypothesized that if pancreatic β cells are protected from over release of insulin in response to high blood glucose concentrations, β cell mass and function may be preserved for a long term benefit.

We previously reported that geniposide was a novel agonist of glucagon-like peptide-1 receptor (GLP-1R) and protected neurons from oxidative stress-induced damage by activating GLP-1R [9–13]. Interestingly, GLP-1R also plays an important role in β-cell function and insulin secretion [14]. Several studies have demonstrated that geniposide inhibit lipotoxicity-induced pancreatic β-cell apoptosis and prevented hIAPP-induced cytotoxicity in INS-1E β cell line [15,16]. Geniposide increases acute insulin secretion in response to low and moderately high glucose levels in INS-1 β cells [14]. However, the effect of geniposide on GSIS in response to high concentrations of glucose is unknown. Moreover, the mechanism of action is not well understood. 

Emerging evidence demonstrates that the classic K_ATP_ channel-dependent mechanism of GSIS does not fully explain the effect of glucose on insulin secretion [17,18]. The results of recent studies suggest that the cyclic pathway of pyruvate metabolism is involved in the regulation of insulin secretion [19–21]. We have here postulated for the first time that geniposide induces insulin secretion in the presence of low and moderately high concentrations of glucose via regulating the uptake and metabolism of glucose and intracellular ATP levels. We have also hypothesized that geniposide protect pancreatic β cells from over insulin secretion damage via altering glucose metabolism when a high concentration of glucose occurs. We have further investigated the role of pyruvate carboxylase, the major enzyme of anaplerosis and α-ketoglutarate (α-KG), an important intermediate of tricarboxylic acid cycle (TCA) involved in the regulation of geniposide on GSIS in pancreatic β cells. 

## Materials and Methods

### Cell culture

Rat INS-1 pancreatic β cell line was purchased from CCTCC (China Center for Type Culture Collection). The cells were cultured at 37 °C in a humidified atmosphere containing 5% CO2. The culture medium was RPMI medium 1640 containing 11 mM glucose and supplemented with 10% FBS, 10 mM HEPES, 100 U/ml penicillin, 100 µg/ml streptomycin, 2 mM L-glutamine, 1 mM sodium pyruvate and 50 μM mercaptoethanol. The culture medium was replaced every second day, and cells were passaged once a week following trypsinization. 

### Insulin secretion assay

To determine the effect of geniposide on GSIS, INS-1 cells were seeded onto 12-well plates and cultured for 24 hours. Then, the cells were washed two times with Krebs-Ringer bicarbonate buffer (KRBB, 129 mM NaCl, 4.8 mM KCl, 1.2 mM MgSO_4_, 1.2 mM KH_2_PO_4_, 2.5 mM CaCl_2_, 5 mM NaHCO_3_, 0.1% BSA, 10 mM HEPES, (pH 7.4) and 2.8 mM glucose) and starved for 2 hours in KRBB. The cells were incubated in fresh KRBB containing different concentrations of geniposide for 1 hour in the presence or absence of different concentrations of glucose. The supernatants were collected to measure insulin concentration using commercial kits (Linco Research, Inc., St Charles, MO) according to the kit’s instructions.

### Glucose uptake and metabolism

To determine the effect of geniposide on glucose uptake and metabolism, INS-1 cells were seeded onto 6-well plates. After overnight incubation, the cells were washed once with KRBB and starved for 2 hours in fresh KRBB in the presence or absence of 10 μM geniposide. The buffer was then replaced with KRBB containing various concentrations of glucose (5.5, 11 or 33 mM). After 20 minutes of incubation, the buffer was collected for the measurement of glucose concentration, which was used to calculate glucose uptake as reported previously. The cell lysates were used to determine ATP content. Glucose concentration in the buffer was measured using a glucose assay kit according to protocol supplied by the manufacturer (Bioversion, Mountain View, CA). The content of ATP in cell lysates was measured using ATP bioluminescence assay kits according to the manufacturer’s instructions (Roche, Mannheim, Germany).

### Plasmid construction and transfection

The shRNA plasmids for pyruvate carboxylase gene were constructed as described elsewhere [22]. Briefly, based on the rat pyruvate carboxylase (NM_012744.2) gene, an oligonucleotide sequence of pyruvate carboxylase shRNA was selected to knock down pyruvate carboxylase expression in INS-1 cells. The rat pyruvate carboxylase-specific shRNA [5’-TGAAGCCTACCTTATTGGC-3’(#1), 5’-GCTGGAAGAGAATTACACC-3’(#2), 5’-CCAGAAGTTGCTACATTAC-3’(#3), and 5’-GTCGCACTAAATACTCA CT-3’(#4)] plasmids were provided by GeneCopoeia Inc (Frederick, MD, USA). Constructed plasmids were transfected into INS-1 cells using FuGENE HD transfection reagent (Roche, CA, USA). 

### Real-time PCR

INS-1 cells were collected and total mRNA was isolated using Qiagen Rneasy Mini Kit (74104, Qiagen, Hilden, Germany), following manufacturer’s instruction. The concentration and integrity of mRNA was evaluated using spectrophotometer (Thermo Fischer Scientific, USA). The primers for rat pyruvate carboxylase gene (sense: 5′- GACCTTGCACATCAAAGCCC-3′; anti-sense 5′- CTCCATGGGCGAAGTCACC-3′), rat β-acin gene (sense: 5′- CACCCGCGAGTACAACCT TC-3′; anti-sense 5′- CCCATACCCACCATCACACC-3′). Quant One Step qRT-PCR Kit was purchased from Tiangen (Tiangen Biotech, Beijing, China). Real-time PCR was performed by Rotor-Gene Q 5plex (Qiagen, CA, USA) in a 25 μl final volume containing (12.5 μl 2 × Quant One Step SYBR qRT-PCR Master mix, 1.25 μl Hotmaster Tag Polymerase at 2.5 U/μl, 0.2 μl Quant RTase, 2 μl sense and anti-sense primers for the final concentrations of 200 nM, 2.5 μl RNA templates for a final quantity of 50 ng, and RNase-free ddH_2_O). First strand cDNA synthesis was performed on 50°C for 30 min. Cycling conditions were: An initial denaturation at 94°C for 5 min, denaturation at 95°C for 30 s, annealing at 54°C for 1 min and extension at 72°C for 1 min. PCR reaction was repeated 40 cycles. Reverse transcriptase PCR conditions was optimized and pyruvate carboxylase mRNA levels in the samples were calculated according to have been normalized against an internal house-keepoing gene, β-actin (Data not shown).

### Immunoblotting

The INS-1 cells were washed with cold PBS and lysed in a lysis buffer containing 20 mM Tris-HCl (pH 7.5), 150 mM NaCl, 1 mM EDTA, 1% (v/v) Triton X-100, 0.1% sodium dodecyl sulfate, protease inhibitors (aprotinin, 30 µg/ml; leupeptin, 4 µg/ml; pepstatin, 2 µg/ml; and phenylmethyl sulfonyl fluoride, 10 µg/ml), 1 mM Na_3_VO_4_, and 2.5 mM Na_4_P_2_O_7_. Lysates were sonicated and measured for protein concentration. The samples were stored at -80°C for other analyses. An aliquot of 10-20 μg protein from each cell extract was loaded on a 10% SDS-PAGE gel. After electrophoretic separation, proteins were transferred to polyvinylidene difluoride (PVDF) membrane. Primary and secondary antibodies (Santa Cruz, San Diego, CA, USA ) were diluted in a blocking solution and incubated with the membrane for indicated times as described previously [11]. Excess antibody was washed off with 20 mM Tris-buffered saline containing Tween-20 (TBST, 20 mM Tris, 150 mM NaCl and 0.1% Tween 20; pH 7.5). Immunoreactivity was detected using enhanced chemiluminescence (ECL) western blotting kit (Amersham Pharmacia Biotech AB, Uppsala, Sweden). Bands were analyzed by densitometric scanning using the Quantity One software (BioRad, Munich, Germany).

### Statistical analysis

All statistical analyses were conducted using the software of Origin version 8.0 (OriginLab Corporation, MA). Data were analyzed using one-way ANOVA followed with the Tukey’s post-doc test or two-way ANOVA followed with the Bonferroni’s post-hoc test for the differences among the treatment means, where p < 0.05 was considered significant. Results are presented as means ± SD. 

## Results

### Regulation of geniposide on acute GSIS depends on glucose levels

**Figure 1:**
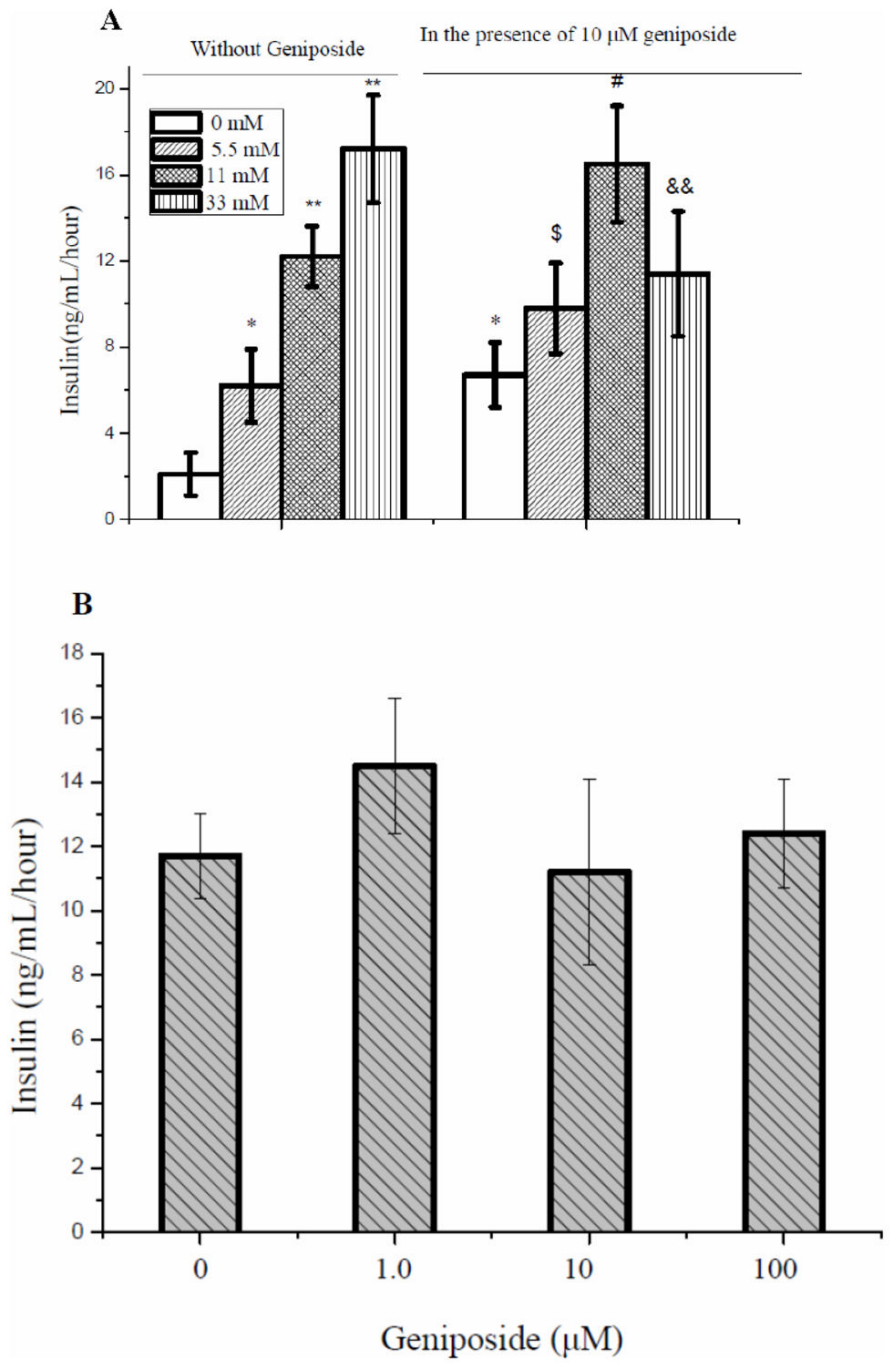
Geniposide differentially regulated insulin secretion in β-cells at different glucose concentrations. A: Insulin secretion in INS-1 cells in response to 0, 5.5, 11, or 33 mM of glucose in the presence or absence of 10 μM geniposide. * P < 0.05, ** P < 0.01 vs vehicle; $, # P < 0.05, && P < 0.01 vs the same glucose concentrations without geniposide. B: Insulin secretion in INS-1 cells incubated at indicated geniposide concentrations in the presence of 35 mM KCl. After the cells were washed twice with KRBH buffer and starved for 2 hours. The indicated concentrations of geniposide and 35 mM KCl were added and continued to incubate for one hour. The supernatant was collected to determine the content of insulin using the commercial ELISA kits. Data are means ± SD from at least three representative experiments (n = 3, two wells for each replicate).

As expected, glucose dose-dependently stimulated insulin secretion in INS-1 cells in the absence of geniposide. Interestingly, in the presence of geniposide at a concentration of 10 μM GSIS was further increased in response to glucose at 5.5 or 11 mM. but decreased in the presence of a high concentration of glucose (Figure 1A). Geniposide increased insulin secretion by 58% and 38% when INS-1 cells were exposed to 5.5 mM and 11 mM of glucose and in contrast reduced insulin secretion by 35% when stimulated with 33 mM of glucose as compared with the cells treated with the same concentrations of glucose alone.

To further explore whether the effect of geniposide on insulin secretion is glucose dependent, we treated INS-1 cells with 35 mM KCl in the presence of various concentrations of geniposide. As shown in Figure 1B, in the absence of glucose, geniposide at concentrations up to 100 μM did not show any significant effect,suggesting that the regulation of geniposide on insulin secretion is glucose dependent.

### Effect of geniposide on glucose uptake and intracellular ATP levels in pancreatic β cells

**Figure 2:**
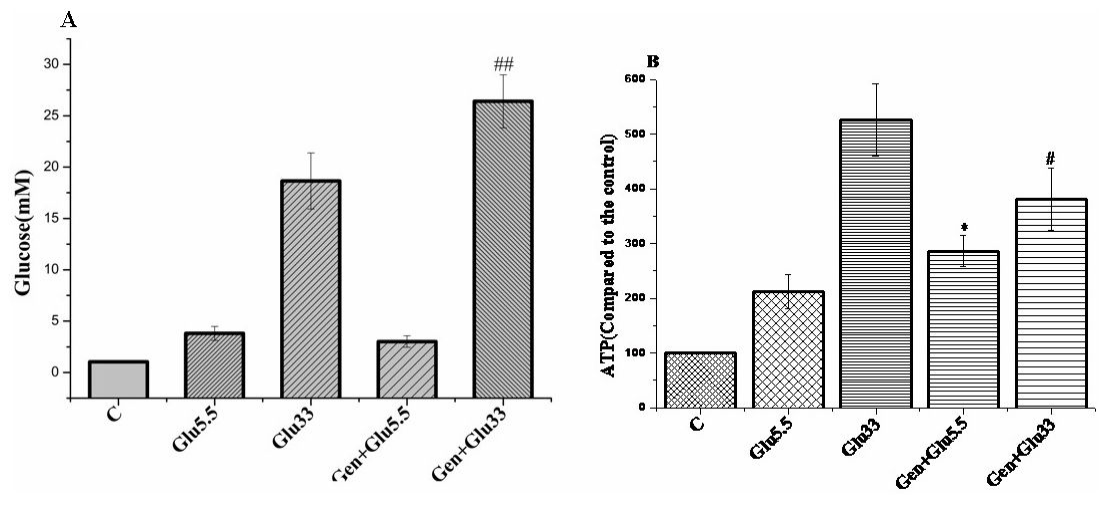
Effect of geniposide on glucose uptake and ATP production in INS-1 cells. INS-1 cells were seeded onto 6-well plate. After overnight incubation, the cells were washed two times with KRBH buffer and starved for 2 hours in KRBH buffer. Then, different concentrations of glucose were added in the buffer and incubated for 20 minutes in the presence or absence of 10 μM geniposide. Glucose concentration in the buffer was measured and the uptake of glucose was determined by the difference of glucose concentrations in the buffer after incubation relative to pre-incubation. The intracellular content of ATP was measure in cell lysates using ATP bioluminescence assay kits according to the manufacturer’s instructions. Data are means ± SD from three representative experiments (n = 3, two wells for each replicate). ^*^ P <0.05 vs 5.5 mM glucose treatment; ^#^ P<0.05, ^##^ P < 0.01 vs 33 mM glucose treatment.

Insulin secretion by pancreatic β cells is influenced by a variety of effectors, with glucose being the primary and most important stimulus, which requires the uptake and metabolism of glucose to increase intracellular ATP concentration and ATP/ADP ratio [23]. Results in Figure 1A and B suggest that regulation of insulin release by geniposide in INS-1 β cells depends on glucose. To verify a possibility that geniposide influenced GSIS in INS-1 cells through altering the intracellular ATP levels and the ration of ATP/ADP, we measured glucose uptake in INS-1 cells treated with 10 μM of geniposide in the presence of 5.5 or 33 mM of glucose. Geniposide increased glucose uptake at the low concentration of glucose but decreased glucose uptake at the high concentration of glucose compared to cells treated with the same concentrations of glucose alone (Figure 2A). In accordance with the effect on glucose uptake, geniposide increased intracellular ATP concentration in INS-1cells at 5.5 mM glucose but decreased the ATP content at 33 mM glucose (Figure 2B). 

### Pyruvate carboxylase plays an important role in the regulation of geniposide on GSIS

**Figure 3:**
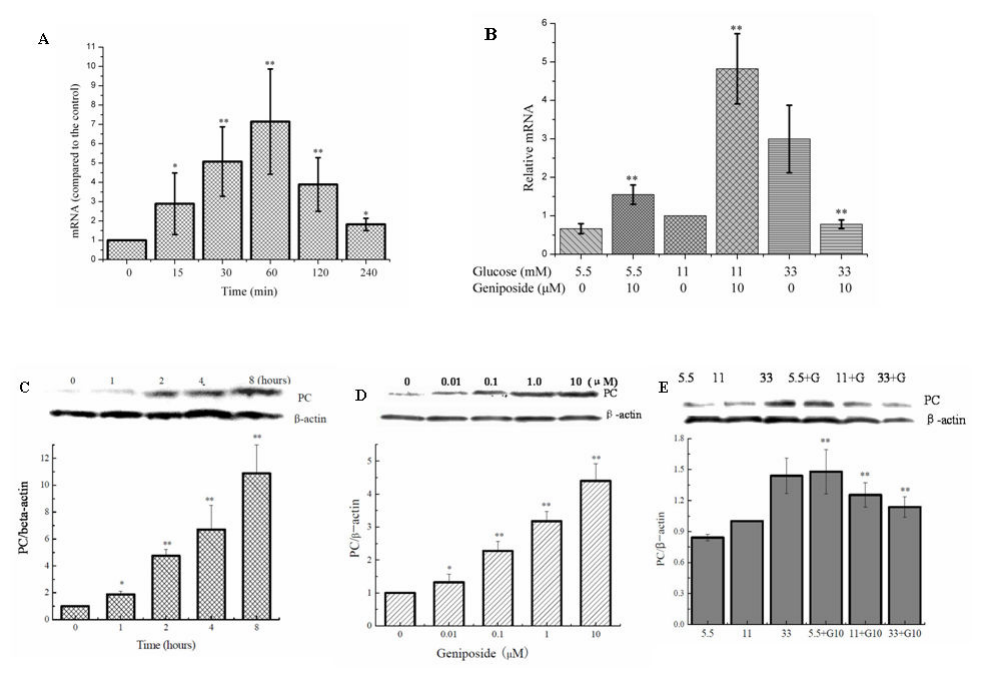
Geniposide regulated the expression of pyruvate carboxylase gene in INS-1 cells. A: Effect of treatment with 10 μM geniposide on the mRNA levels of pyruvate carboxylase gene, *, ** P < 0.05 , P < 0.01 vs the control. B: Effect of 10 μM geniposide treatment for 1 hour on the mRNA levels of pyruvate carboxylase gene in the presence of different concentrations of glucose,* P < 0.05, **, P < 0.01 vs the same glucose concentrations without geniposide. C: Treatment with 10 μM geniposide increases the protein levels of pyruvate carboxylase in INS-1 cells in a time-dependent manner, * P < 0.05, ** P < 0.01 vs vehicle. D: Exposure of geniposide for 2 hours increased the protein levels of pyruvate carboxylase in a concentration-dependent manner, * P < 0.05, ** P < 0.01 vs vehicle. E: Exposure of 10 μM geniposide for one hour differently altered pyruvate carboxylase protein levels in INS-1 cells in the presence of low and high glucose concentrations, ** P < 0.01 vs the same glucose concentration without geniposide. Data are means ± SD from at least three independent experiments.

Pyruvate carboxylase is expressed at high levels, amounting up to 0.4% of total proteins in pancreatic islets, β cells, and INS-1 cell line, respectively, but at low levels in non-β cells of the islets [24,25]. In β cells, a large portion of pyruvate is derived from glucose metabolism. Approximately half of pyruvate enters the mitochondria where pyruvate is carboxylated by pyruvate carboxylase to form oxaloacetate. The rate of pyruvate carboxylation correlates with glucose concentration and the rate of insulin secretion in isolated pancreatic islets [25,26]. As shown in Figure 3, the current study showed that geniposide acutely increased the levels of mRNA and protein of pyruvate carboxylase in a time- and dose-dependent manner in the presence of 5.5 and 11 mM glucose. However, when INS-1 cells were cultured with 33 mM glucose, accompanied with the inhibition on mRNA levels of pyruvate carboxylase, incubation with 10 µM geniposide for one hour also decreased the expression of pyruvate carboxylase protein as compared with cells treated the same concentration of glucose alone. 

**Figure 4:**
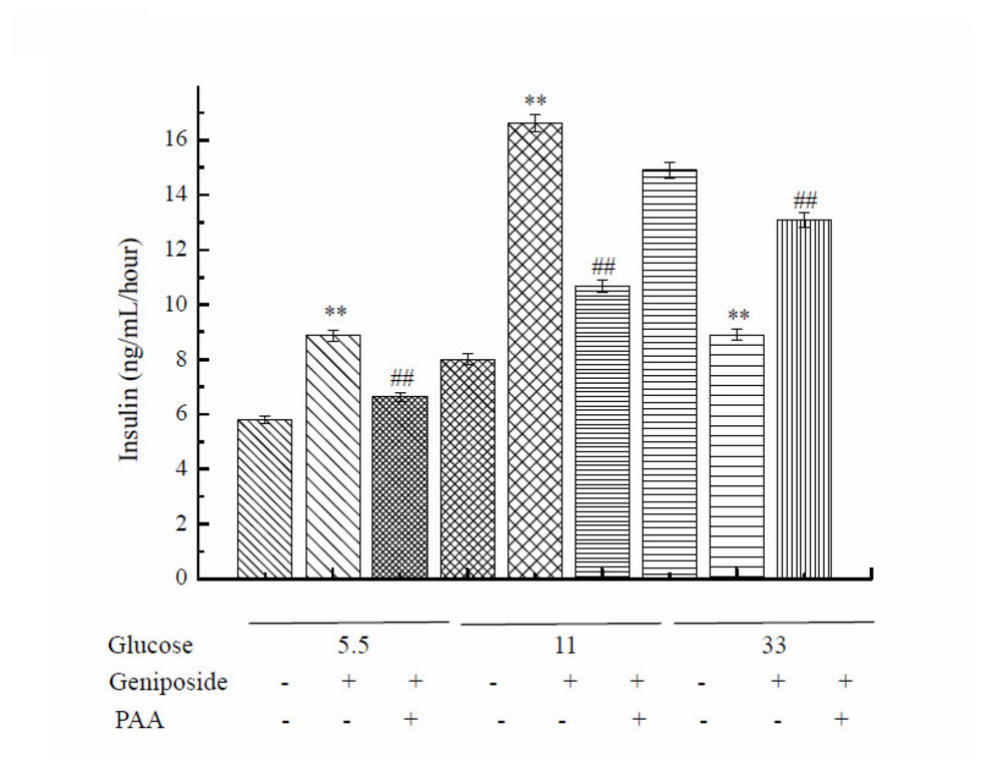
Pyruvate carboxylase inhibitor PAA abolished the effect of geniposide on GSIS in INS-1 cells. INS-1 cells were seeded onto 6-well plate. After overnight incubation, the cells were washed two times with KRBH buffer and starved for 2 hours in KRBH buffer. After that, the cells were pretreated with 5 mM PAA for 30 minutes, and then, different concentrations of glucose with or without 10 μM geniposide were added in the buffer and incubated for 1 hour. The supernatant was collected to determine the content of insulin using commercial ELISA kits. Data are means ± SD (n = 3, two wells for each replicate). ** P < 0.01 vs the same glucose concentration, ## P< 0.01 vs the same glucose concentration with PAA.

**Figure 5:**
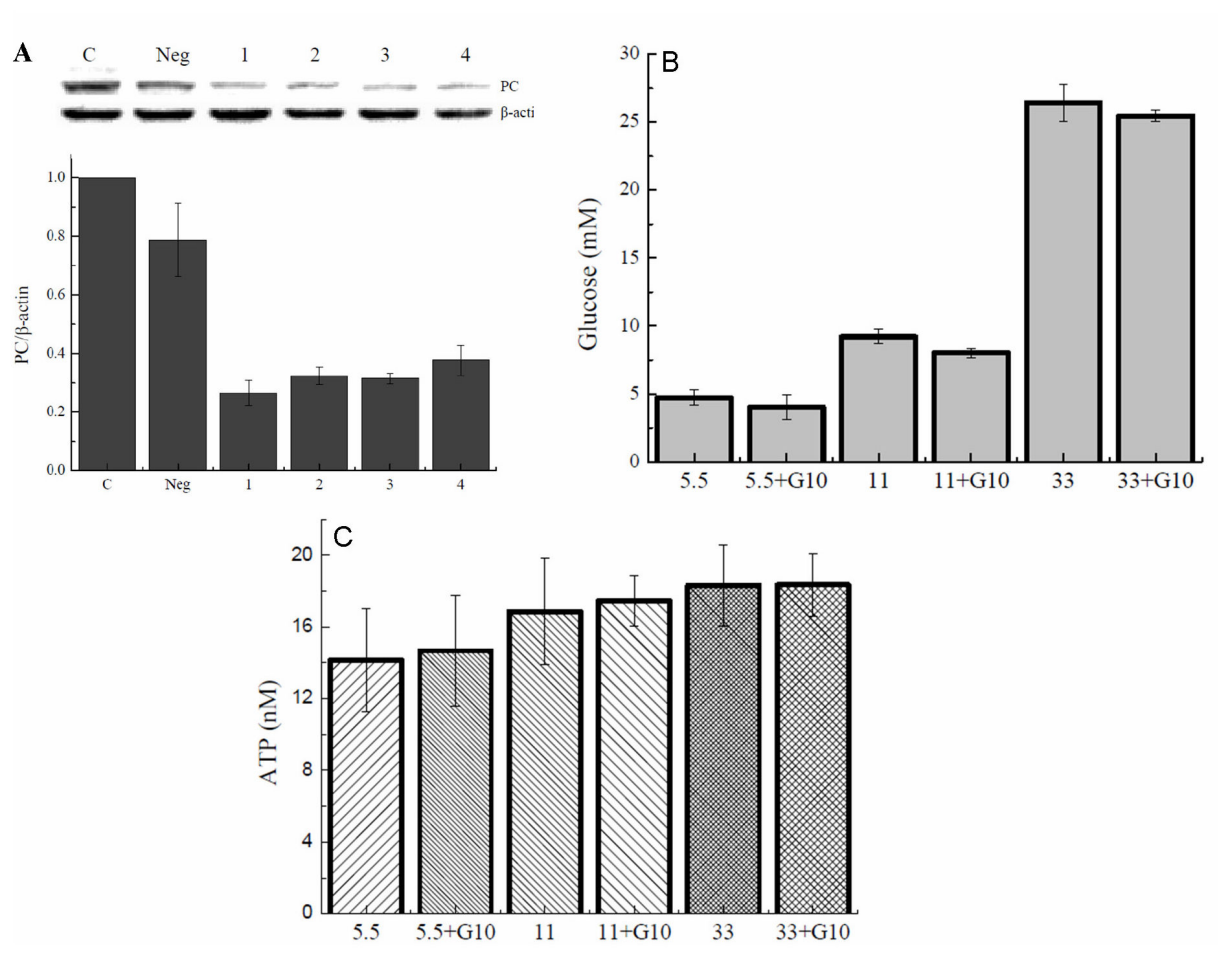
Effect of pyruvate carboxylase shRNA interference on the regulation of geniposide on the uptake and metabolism of glucose in INS-1 cells. A: The interfering efficiency of shRNA for pyruvate carboxylase gene was determined by western blot. B: Interference on pyruvate carboxylase expression reversed the influence of geniposide on glucose uptake in the presence of low or high concentration of glucose. C: Interference on pyruvate carboxylase expression inhibited the effect of geniposide on ATP production in the presence of low or high concentration of glucose. Data are means ± SD from three representative experiments.

To verify the regulatory role of pyruvate carboxylase in geniposide-modulated GSIS, we incubated the INS-1 cells for 1 hour with a pyruvate carboxylase inhibitor PAA prior to geniposide treatment. The pre-incubation with PAA reversed the effect of geniposide on GSIS (Figure 4). In further experiment, we transfected the INS-1 cells with pyruvate carboxylase shRNA. It was found that the effect of geniposide on pyruvate carboxylase expression was abolished (Figure 5A). Consequently, the effect of geniposide on the glucose uptake and intracellular ATP levels were attenuated (Figure 5B and 5C). These data suggest that pyruvate carboxylase might have played a key role in the regulation of GSIS by geniposide, possibly via modulating the glucose uptake and metabolism, leading to the alteration of ATP production and the ratio of ATP/ADP in INS-1 cells. 

### Effect of geniposide on α-ketoglutarate production in INS-1 cells

**Figure 6:**
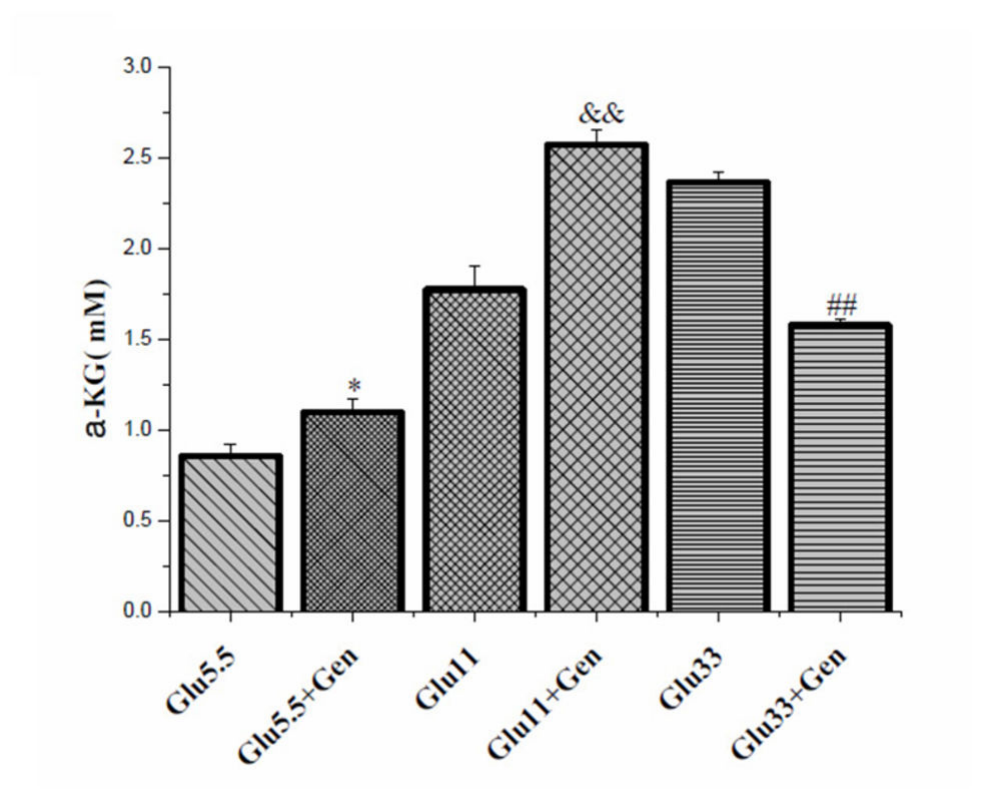
Geniposide regulated α-ketoglutarate production in the presence of different concentrations of glucose in INS-1 cells. INS-1 cells were seeded and cultured overnight. Next day, cells were washed twice with KRBB and starved for 2 hours in KRBB in the presence or absence of 10 μM geniposide. The media were replaced with fresh complete media containing 5.5, 11, or 33 mM glucose with or without 10 μM geniposide. Following one hour incubation, the cells were washed twice with cold PBS. The cells were lysed with lysis buffer and the lysates were used to determine the content of α-ketoglutarate using a commercial kit according to the kit’s instructions. Data are means ± SD from three representative experiments. *, p < 0.05 vs the group of 5.5 mM glucose alone, &&, p < 0.01 vs the group of 11 mM glucose alone, and ##, p < 0.01 vs the group of 33 mM glucose alone respectively.

Several studies have shown that in pancreatic β cells, a significant amount of pyruvate enters the TAC cycle via carboxylation, augmenting the production of the cycle intermediates by anaplerosis [26,27]. Surplus of the intermediates are exported out of the mitochondria and triggers insulin release by getting into the insulin secretory granules [28]. In accordance with the effect on pyruvate carboxylase protein expression and GSIS, geniposide exerted a regulatory effect on the production of the intermediates of glucose metabolism, for example, α-ketoglutarate (Figure 6). Geniposide increased α-ketoglutarate concentration at 5.5 or 11 mM glucose but decreased α-ketoglutarate concentration at 33 mM glucose in INS-1 cells relative to the cells treated with the same concentrations of glucose alone. 

### Effect of geniposide on the function of β-cells treated with high levels of glucose

**Figure 7:**
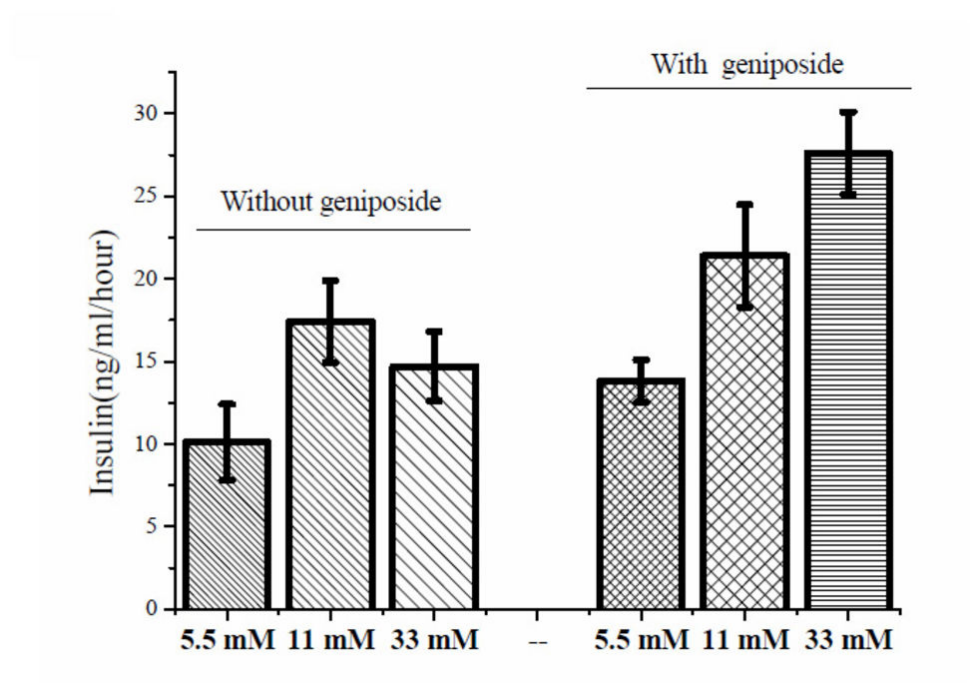
Pre-incubation with geniposide improved the impairment of GSIS in response to a high glucose concentration in INS-1 cells. After INS-1 cells were incubated with 33 mM glucose for 24 hours in the presence or absence of 10 μM geniposide, the cells were washed once and the media were replaced with fresh KRBB containing 5.5, 11, or 33 mM glucose. After 1 hour incubation, the media were collected for the determination of insulin content with commercial ELISA kits. Data are means ± SD (n = 3) .

We observed that acute treatment of INS-1 cells with geniposide decreased GSIS in INS-1 cells in response to the stimulation of a high glucose concentration. To further investigate the chronic influence of geniposide on GSIS, INS-1 cells were cultured at 33 mM glucose and 10 µM geniposide for 24 hours prior to the stimulation of insulin secretion by different glucose concentrations. In line with the previous reports [4,5], insulin secretion was impaired in INS-1 cells cultured at 33 mM glucose for 24 hours. However, in the presence of geniposide, this impairment was markedly improved (Figure 7). So, it was obviously, acute exposure to high glucose increased insulin secretion whereas chronic exposure decreased insulin in normal cultured pancreatic cells. The present study shows that geniposide has opposite effects and protects pancreatic cells from over release of insulin-induced damage and preserve cells for insulin secretion functions. 

## Discussion

The present study demonstrated that geniposide enhanced GSIS in the presence of low or moderately high concentrations of glucose, in line with a previous study [14]. Interestingly and for the first time, to our knowledge, it was noticed that geniposide increased glucose uptake and intracellular ATP content in pancreatic β-cells cultured with low or moderately high concentrations of glucose. The increased pyruvate carboxylase expression and α-ketoglutarate production by geniposide indicates that geniposide might have increased substrates available for the TCA cycle and/or the efficiency of TCA cycle, leading to the increase of intracellular ATP. Although geniposide may also affect the utilization of ATP, the increased glucose uptake by geniposide indicates that the increase of intracellular ATP content is, at least in part, a result of enhanced production of ATP, which is a key driving factor for insulin release[29]. Further experiments demonstrated that geniposide increased the gene and protein expressions of pyruvate carboxylase in a time- and dose-dependent pattern. The data of the present study indicate a sequence of events in which geniposide increased pyruvate carboxylase expression, speeded up the uptake and metabolism of glucose, promoted the production of ATP and α-ketoglutarate, and ultimately improved the coupling between glucose metabolism and insulin secretion in pancreatic β-cells in response to low or moderately high concentrations of glucose. 

However, glucotoxicity occurs in β-cells when the cells are exposed to high concentrations of glucose for an extended period. In humans, when individuals initially experience insulin resistance, a compensatory process of β-cells is triggered to releasing more insulin into the bloodstream to match the increased demand of insulin for glucose disposal. This process gradually exhausts β-cells and eventually result in a situation that some of β-cells are no longer functional to produce and secrete insulin, shifting blood hyperinsulemia to hypoinsulemia [30,31]. Up to this stage, the patients lose the ability to maintain normal blood glucose levels and start to show hyperglycemia and other diabetic symptoms. As reported, glucotoxicity together with lipotoxicity contributes to the deterioration of insulin secretion over the years following the diagnosis of T2DM [32]. Evidently, the preservation of β-cell function and mass becomes critical for the prevention and treatment of insulin resistance and T2DM.

Diabetes research and product development have long been focused on the reverse or improvement of diabetic conditions through enhancing insulin secretion and/or improving peripheral insulin sensitivity. However, it is increasingly understood that the continuous exposure to high levels of blood glucose gradually leads to diminution or complete loss of functions and even cell death of β-cells [8,33,34]. It is, therefore, reasonably thinking that if pancreatic β cells are protected or preserved in response to hyperglycemia, a long-term benefit for glucose control may be achieved. This notion was supported by the findings of the present study. i.e., the impaired GSIS of INS-1 β cells by 24-hour exposure to a high concentration of glucose (33 mM) was reversed by geniposide. This finding suggests that geniposide may have a protective role against glucotoxicity in pancreatic β cells. 

To understand how geniposide protected β cells from glucotoxicity, we have conducted further experiments in INS-1 cells. An interesting observation is that geniposide exerted an acute inhibitory effect on glucose uptake and ATP content in pancreatic β-cells when the cells were challenged acutely with a high concentration of glucose. To explain the mechanism of action, we have proposed that when pancreatic β cells are acutely challenged with a high glucose concentration, geniposide rapidly suppressed pyruvate carboxylase expression and thus prevent over amount of pyruvate entering the TCA cycle, consequently protect the β cells from glucotoxicity,leading to the reductions of ATP and α-ketoglutarate productions as compared with cells treated with a high concentration of glucose alone. The ATP-sensitive potassium channel senses metabolic changes, thereby coupling metabolism to electrical activity and ultimately to insulin secretion [29]. The attenuation of ATP prodcution by geniposide at an acute high glucose stimulation resulted in a decrease of ATP/ADP ratio, leading to the opening of ATP-sensitive potassium channels [35,36] and the reduction of insulin secretion compared to the cells challenged with the high concentration of glucose. Thus, attenuation of GSIS by geniposide at acute challenge of high glucose concentrations could be partially explained by a relative reduction of glucose-derived intracellular ATP levels. As mentioned earlier, insulin secretion is controlled through both the K_ATP_ channel dependent- and independent-pathways. Accumulating evidence suggests that anaplerosis, the biosynthesis of citric acid cycle intermediates, is also involved in the regulation of insulin secretion in addition to the K_ATP_ channel dependent pathway [37–39]. Pyruvate carboxylase is the key anaplerotic enzyme of the pyruvate cycling and has been shown to play a central role in insulin secretion in the pancreatic β cells of rodents and clonal insulin-producing cell lines [25,40–43]. A number of studies have demonstrated that pyruvate is cycled through pyruvate carboxylase in rodent clonal β cell lines [39,44] and mouse pancreatic islets [45]. It is also reported that the rate of pyruvate cycling through pyruvate carboxylase is proportional to the capacity of GSIS in various INS-1 cell lines [46]. Hasan et al. recently used RNAi knockdown technology to produce a series of cell lines derived from the rat insulinoma cell line to express different levels of pyruvate carboxylase. In these cell lines, the insulin release stimulated by glucose and other metabolizable insulin secretagogues was inhibited in proportion to the severity of pyruvate carboxylase knockdown [47]. The severe knockdown of pyruvate carboxylase caused a metabolite crossover point in glucose-stimulated β cells, increased pyruvate plus lactate and decreased malate and citrate, being consistent with blocking the pyruvate carboxylase activity [47]. Moreover, the “pyruvate/isocitrate cycle” involving the export of citrate and isocitrate from the mitochondria to cytosol via citrate/isocitrate carrier, and pyruvate/citrate cycle directly regulates insulin secretion rather than through a consequent change of ATP/ADP ratio [35,36]. During this process, isocitrate (or citrate) is converted to α-ketoglutarate by a cytosolic NADP-dependent isocitrate dehydrogenase and recycled to oxaloacetate or pyruvate by one of several possible cytosolic or mitochondrial pathways. It has been well documented that the intermediates (coupling factors) of pyruvate cycling, including pyruvate, malate, citrate, isocitrate, ATP, α-ketoglutarate, and NADPH serve as the second messengers to insulin release[35,36]. It has also been shown that regulation of pyruvate cycling by glucose is perturbed in T2DM [48,49]. Our results, together with the published information, demonstrate that geniposide regulates GSIS through modulating glucose metabolism and TCA cycle, and PC is the key enzyme involved in this regulation. 

It is known that glucose uptake in pancreatic β-cells is regulated by glucose transporter 2 (GLUT-2). The low affinity of GLUT2 allows glucose sensing and control the rate of glucose uptake by the cells form the blood stream. Thus, the expression of GLUT-2 is more pronounced when glucose concentration is high in the culture media and increases as culturing continues [50]. We observed that geniposide increased in a dose-dependent manner the expression of GLUT2 at 11 mM of glucose in INS-1 β cells (data not shown). As not determined in the present study, it is not known whether geniposide regulates the expression of GLUT2 differentially at low and high glucose concentrations and at acute and chronic high glucose challenges. In addition, further investigations are needed to determine whether geniposide regulates glucose uptake and metabolism and pyruvate carboxylase expression through GLP-1R. It is also interesting to know if the observed effects of geniposide in INS-1 cells represent primary β cell function. These aspects are the limitation of the current study.

We previously reported that pretreatment of β cells with geniposide inhibited the early-stage apoptosis induced by palmitate, and antagonized the cytotoxicity of human islet amyloid polypeptide (hIAPP) [15,16]. Many *in vivo* studies have demonstrated beneficial effects of geniposide on diabetes in diet-induced diabetic rodent models [51]. Studies in diabetic mice showed that geniposide was an effective hypoglycemic agent by inhibiting the activities of hepatic glycogen phosphorylase (GP) and glucose-6-phosphatase (G6Pase) [52]. Here we observed that geniposide, instead of taxing β cells to maximize its insulin secretion in order to lower blood glucose, preserved pancreatic β cells under the stimulation of a high or very high glucose concentration by modulating glucose uptake and metabolism. 

In conclusion, geniposide is promising as a naturally-occurring agent to prevent or delay the onset and progression of diabetes, due to its capacity to preserve pancreatic β cells from exhaustion and damage resulted from prolonged and over insulin secretion and glucotoxicity under high glucose burden. There is potential to use geniposide as a β cell protectant in humans who are at a high risk for developing type 2 diabetes and those being diagnosed to have pre- or early diabetes.
