# Correction: Geniposide Regulates Glucose-Stimulated Insulin Secretion Possibly through Controlling Glucose Metabolism in INS-1 Cells 

**DOI:** 10.1371/annotation/129e1764-d44a-4b81-8b1d-ba8bce898a7b

**Published:** 2014-01-06

**Authors:** Jianhui Liu, Lixia Guo, Fei Yin, Yonglan Zhang, Zixuan Liu, Yanwen Wang

The number of the grant from the National Natural Science Foundation of China is incorrect. The correct grant number is: 81373459. 

